# Contaminated Turmeric Is a Potential Source of Lead Exposure for Children in Rural Bangladesh

**DOI:** 10.1155/2014/730636

**Published:** 2014-08-24

**Authors:** Kelsey Gleason, James P. Shine, Nadia Shobnam, Lisa B. Rokoff, Hafiza Sultana Suchanda, Md Omar Sharif Ibne Hasan, Golam Mostofa, Chitra Amarasiriwardena, Quazi Quamruzzaman, Mahmuder Rahman, Molly L. Kile, David C. Bellinger, David C. Christiani, Robert O. Wright, Maitreyi Mazumdar

**Affiliations:** ^1^Department of Environmental Health, Harvard School of Public Health, 677 Huntington Avenue, Boston, MA 02115, USA; ^2^Jefferson Medical College, 1025 Walnut Street, Philadelphia, PA 19107, USA; ^3^Oregon State University, 15 Milam, Corvallis, OR 97331, Bangladesh; ^4^Mount Sinai School of Medicine, 17 East 102nd Street, New York, NY 10029, USA; ^5^Oregon State University, 15 Milam, Corvallis, OR 97331, USA; ^6^Department of Neurology, Fegan 11 Neurology, Boston Children's Hospital, 300 Longwood Avenue, Boston, MA 02115, USA

## Abstract

*Background.* During the conduct of a cohort study intended to study the associations between mixed metal exposures and child health outcomes, we found that 78% of 309 children aged 20–40 months evaluated in the Munshiganj District of Bangladesh had blood lead concentrations ≥5 *µ*g/dL and 27% had concentrations ≥10 *µ*g/dL. *Hypothesis.* Environmental sources such as spices (e.g., turmeric, which has already faced recalls in Bangladesh due to high lead levels) may be a potential route of lead exposure. * Methods.* We conducted visits to the homes of 28 children randomly selected from among high and low blood lead concentration groups. During the visits, we administered a structured questionnaire and obtained soil, dust, rice, and spice samples. We obtained water samples from community water sources, as well as environmental samples from neighborhood businesses. *Results.* Lead concentrations in many turmeric samples were elevated, with lead concentrations as high as 483 ppm. Analyses showed high bioaccessibility of lead. *Conclusions.* Contamination of turmeric powder is a potentially important source of lead exposure in this population.

## 1. Introduction

Lead poisoning remains a major public health problem, particularly for young children in developing countries. Several epidemiological investigations have shown a high prevalence of elevated blood lead concentrations among Bangladeshi children living in large urban and industrial centers [1–3]. Children in rural Bangladeshi communities may also be at risk of exposure to lead through the continued use of leaded gasoline in rural areas [2], poorly developed waste management systems, and cottage industries that are increasingly found in rural settings. The harmful effects of lead are exacerbated by poor nutrition and micronutrient deficiencies, also highly prevalent in rural Bangladesh. The purpose of this study was to evaluate potential sources of lead exposure among children in a rural community in Bangladesh who had been identified in an ongoing cohort study as having high blood lead concentrations.

## 2. Methods

### 2.1. Subject Selection

This research was conducted in Sirajdikhan, located in the Munshiganj District in Bangladesh, a primarily rural and agricultural area largely devoted to the cultivation of rice and potatoes. Sirajdikhan is located 29.3 km southwest of Dhaka city, with a total population of 1.4 million and population density of 1,439 persons per km [2]. The literacy rate is 56.4% among males and 55.7% among females. Approximately 12% of the district is considered urbanized (having an area around a central place with improved communication, electricity, water supply, sanitation, and nonagricultural occupations) [4]. Emerging local industries include brick kilns, textile weaving/dyeing, and carpentry/woodworking.

Participants were children who are currently enrolled in an ongoing population-based prospective birth cohort study designed to investigate the association between prenatal arsenic exposure and neurodevelopment in children. Participants were recruited from areas in which Dhaka Community Hospital (DCH) operates rural health clinics. After high blood lead concentrations were identified during an ongoing study, we selected 30 participants for environmental exposure assessment. Participants were grouped into quartiles according to blood lead concentrations, and 15 subjects from both the highest and lowest quartiles were selected for participation through random digit assignment. Twenty-eight families agreed to participate in exposure assessment activities.

Details of the recruitment strategy, eligibility criteria, and sample collection from pregnant women and newborns have been published previously [5, 6]. All children in the birth cohort study were eligible for followup studies, and DCH-trained health care workers invited their families to participate. Informed consent was provided by the parents of all participants before enrollment. Boston Children's Hospital's Committee for Clinical Investigation ceded review of this study to the Harvard School of Public Health (HSPH). This study was approved by the Human Research Committees at HSPH and DCH.

### 2.2. Blood Lead Measurements

Lead concentrations were measured in blood samples collected from fingerstick blood collected at 20 to 40 months of age, with a subset confirmed with samples obtained via venipuncture. Fingerstick blood lead concentrations were measured using portable LeadCare II instruments (Magellan Diagnostics, Billerica, MA, USA) that have a reportable range of 3.3–65 *μ*g/dL. Before testing, study staff washed the children's hands to avoid lead contamination from the skin. Each child's finger was pricked with a single-use lancet, and a 50 *μ*L capillary tube was filled after discarding the first droplet. Test results were available within 10 minutes. Daily quality-assurance measures were performed according to the user's manual.

Blood samples obtained via venipuncture were collected in trace metal-free tubes for measurement of arsenic, manganese, and lead concentrations, the aims of the primary cohort study. Samples were analyzed for lead at the trace metals laboratory at HSPH in Boston, Massachusetts. Blood samples were first weighed (~1 g) and digested for 24 hours in 2 mL of concentrated nitric acid. These samples were then treated with 1 mL of 30% hydrogen peroxide per 1 g of blood and left overnight. Samples were subsequently diluted to 10 mL with deionized water. Lead concentrations were measured using a dynamic reaction cell-inductively coupled plasma mass spectrometer (DRC-ICP-MS, DRC II, Perkin Elmer). Analyses were performed using an external calibration method using lutetium as an internal standard to account for instrument drift. Continual calibration standards and the use of a standard reference solution (NIST 1643e: Metals in Water) were used to monitor precision and accuracy of the analysis. The final sample concentration used in the results was an average of 5 replicate measurements for each individual samples.

Quality control included analysis, procedural blanks, duplicate samples, spiked samples, and analysis of a certified reference material (NIST 955b: bovine blood for lead) to monitor for contamination, accuracy, and recovery rates. Recovery rates for lead in quality control and spiked samples were 81%–108%, and precision was measured as % relative standard deviation (SD), with a result of less than 3% for lead. The average limit of detection (LOD) was 0.2 *μ*g/dL.

### 2.3. Questionnaires

Trained study staff administered questionnaires that collected medical histories and demographic information. This questionnaire included detailed questions about potential sources of lead exposure including paint, distance to roads and shops, battery recycling, parents' occupations, and children's hand-to-mouth activity.

### 2.4. Environmental Sample Collection

Site visits were conducted at each participant's home, and trained staff collected soil, drinking water, turmeric, and rice samples in trace metal-free 50 mL plastic centrifuge tubes. Three battery recycling/recharging plants were visited, and soil samples were collected from a region of soil closest in proximity to the shop. Soil samples were extracted from an approximately 4′′ × 4′′ × 1′′ area of soil and collected using a plastic trowel. During the ongoing sample collection, samples were stored at room temperature in DCH. Rice, turmeric, and soil samples were sealed with Parafilm and packaged in Coleman coolers for shipment to HSPH. Water samples were analyzed at laboratories at the Bangladesh University of Engineering and Technology (BUET) and Dhaka University.

Soil samples were dried and filtered through a 200 *μ*m sieve to simulate the size of inadvertent particle ingestion. Four grams of soil, turmeric, or rice was transferred into X-ray fluorescence (XRF) analytical cups after thorough homogenization to determine the concentration of total lead in the samples by X-Ray fluorescence (Nilton XL3T, Billerica, MA). Rice samples were ground into a fine powder using a trace metal-free spice grinder. The limit of detection by XRF is approximately 10 ppm. Water samples were tested using atomic absorption spectroscopy (AAS) at laboratories at Dhaka University and the Bangladesh University of Engineering and Technology. The limit of detection by AAS was 0.01 mg/L.

### 2.5. Bioaccessibility Analysis

Lead bioaccessibility in turmeric samples was estimated using the simple bioavailability extraction test (SBET), an in vitro gastric fluid extraction that simulates metal dissolution in the stomach. The SBET was performed following procedures previously established by the US Environmental Protection Agency (EPA) [7, 8]. Following the SBET analysis, lead concentrations were measured using inductively coupled plasma mass spectrometry (ICPMS) to determine the fraction of total lead that was bioaccessible in simulated gastric fluid.

### 2.6. Statistical Analyses

We calculated descriptive statistics for both blood lead measures and selected subject characteristics. We dichotomized blood lead concentrations at different reference levels corresponding to current US and World Health Organization guidelines (i.e., ≥5 *μ*g/dL and ≥10 *μ*g/dL). The government of Bangladesh currently does not have recommended blood lead guidelines for children. Associations between demographic variables and blood concentrations were conducted using Fisher's exact tests. For environmental samples, we calculated descriptive statistics. All analyses were performed in SAS version 9.3 (SAS Institute Inc., Cary, NC, USA).

## 3. Results

In this analysis, we used data from 309 children who provided fingerstick blood samples as of May 1, 2013. Venous blood samples were available for 176 (57%) of these children at the same visit. This sample represents 74% of the planned enrollment from this site for the birth cohort study.  Table 1 presents the demographic characteristics of children and families.

The overall median fingerstick blood lead concentration at approximately the age of 2.5 years was 8.1 *μ*g/dL (range: <LOD-443.0 *μ*g/dL). The mean venous blood lead concentration was 8.0 *μ*g/dL (range 2.0–36.3 *μ*g/dL). There was good correlation between venous blood lead concentrations and fingerstick blood lead concentrations (the Spearman correlation coefficient 0.82, *P* < 0.001). In our sample, 78% of the children tested had fingerstick blood concentrations at or above the current CDC reference level of 5 *μ*g/dL and 27% of the children tested had fingerstick blood lead concentrations at or above the previous CDC action level of 10 *μ*g/dL. Similar distributions were seen among venous blood samples, with 84% of the children having venous blood lead concentrations above 5 *μ*g/dL and 26% having concentrations above 10 *μ*g/dL (Table 2). Sex, parental education level (low education level defined as primary education or less), and BMI were not associated with elevated blood lead concentrations (results not shown).

**Figure 1:**
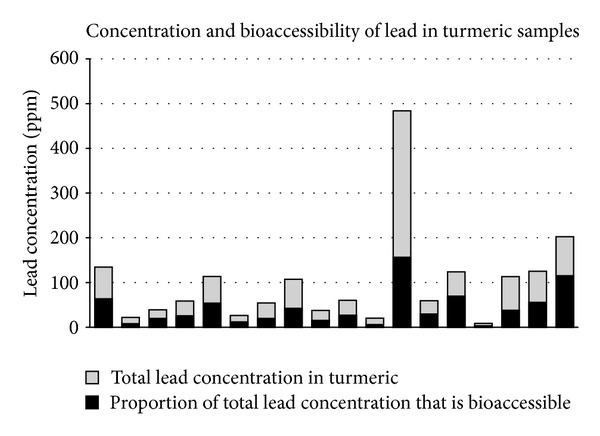
Lead concentration in turmeric samples shown with total lead in turmeric and the fraction that is bioaccessible in simulated gastric fluid. Samples with lead concentrations below the limit of detection were excluded from bioaccessibility analyses.

Lead concentrations varied among our turmeric samples, with a mean of ~80 *μ*g/g and a range of <LOD to 483 *μ*g/g. The average bioaccessibility of lead in turmeric was 42.9%. These results are summarized in Figure 1. Soil samples did not show high concentrations of lead (median = 16.3 *μ*g/g and maximum = 33.6 *μ*g/g). Total chromium concentrations were also measured by XRF to test for the presence of PbCrO_4_ and were not elevated. Only 3 turmeric samples had chromium concentrations above the limit of detection (42 *μ*g/g, maximum = 235 *μ*g/g). No water sample had a lead concentration above the limit of detection. Additionally, no rice sample had a lead concentration above the level of detection. Soil samples from three battery recycling shops showed that the soil inside the shop's walls had elevated lead (mean = 1632 ppm), but outside of the shops the soil lead concentrations were not significantly elevated (mean = 28 ppm).

## 4. Discussion

Almost 80% of children aged 20 to 40 months tested in Munshiganj had blood lead concentrations at or above 5 *μ*g/dL, the reference level currently set by the US Centers for Disease Control and Prevention (CDC), and 26% of the children tested had blood lead concentrations at or above the previous CDC action level of 10 *μ*g/dL. The original intention of the study was to investigate arsenic and manganese toxicity, and blood lead concentration was measured as a potentially important covariate; these high blood lead concentrations were not anticipated.

In our study, turmeric samples had high concentrations of lead, with high levels of bioaccessibility, suggesting that contamination of food spices may be an important source of lead exposure in this setting. The elevated levels of lead in these turmeric samples (mean, ~80 *μ*g/g; range, <LOD to 483 *μ*g/g) in conjunction with an average bioaccessibility of lead in turmeric of 42.9% further support the hypothesis that turmeric is an important source of lead exposure for this cohort.

Previous reports of elevated blood lead concentrations in Bangladeshi children have focused on large cities and industrial areas [1–3]. Sirajdikhan, by contrast, is a primarily rural area, providing evidence that lead poisoning in Bangladesh is not limited to urban centers.

Previous research has identified lead-contaminated foodstuffs originating from Bangladesh and India, further supporting our turmeric findings. A recent study by Saha and Zaman [9] demonstrates the potential for contamination of foodstuffs such as fish and vegetables to occur in urban Bangladeshi settings. In September 2013, the US Food and Drug Administration (FDA) announced voluntary recall by distributors of Pran brand turmeric powder, a Bangladeshi company, due to elevated levels of lead [10]. Additional studies identified the presence of contaminated spices originating from India and Bangladesh in markets in Boston, MA [11–13]. This study builds upon these reports by identifying turmeric as a potential direct exposure route within a vulnerable community.

While this research identifies the presence of lead-contaminated turmeric, the method of contamination is unknown. Uptake of lead from soil into the turmeric is a possible, but unlikely, source of contamination, as previous studies estimate the maximum uptake of lead into the root of the plant to be approximately 10% [14]. Additionally, it is possible that the addition of lead chromate to these samples (to increase the weight and bright color) may be the source of lead contamination. However, we did not find high chromium levels in our turmeric samples, as would be expected with the addition of lead chromate. Further testing and closer inspection of the growing and manufacturing process is needed.

We were not able to assess the amount of turmeric ingested by children, and this is a further limitation of this study. We did not find a strong correlation between turmeric lead concentrations and blood lead concentrations, though we note that the turmeric samples were obtained months after the blood concentrations were measured. We therefore report that the high levels of lead in turmeric are a potential risk to the community.

## 5. Conclusion

There is an urgent need to identify the source of lead exposure in rural communities in Bangladesh. Turmeric, a spice commonly used in Bangladeshi cooking, is a potential source of lead exposure. Further testing is needed to confirm these sources. Once identified, preventative measures can be taken.

## Figures and Tables

**Table 1:** Selected characteristics of study population.

	Sirajdikhan, Munshiganj District, *n* = 309
	Mean (SD)

Age (years)	2.4 (0.2)
BMI (kg/m^2^)	16.7 (2.4)
Hematocrit (*n* = 271)	35.6 (3.9)

	Number (%)

Male sex	158 (51.1)
Place of birth	
Home	115 (37.2)
Clinic or hospital	194 (62.8)
Mother's education	
No primary education	31 (10.0)
Primary education	119 (38.5)
Secondary education	149 (48.2)
Any higher secondary	10 (3.2)
Father's education	
No primary education	57 (18.4)
Primary education	134 (43.4)
Secondary education	102 (33.0)
Any higher secondary	16 (5.2)
Eating nonfood items (pica)	
Yes	146 (47.5)
No	162 (52.4)

**Table 2:** Percentage of children at or above selected blood lead concentrations.

Blood lead concentration (*μ*g/dL)	Fingerstick (*n* = 309)	Venous (*n* = 179)
≥5	78.3	84.1
≥10	26.5	25.6
≥20	1.6	1.0
